# Trends in Positive, Negative, and Neutral Themes of Popular Music From 1998 to 2018: Observational Study

**DOI:** 10.2196/26475

**Published:** 2021-06-24

**Authors:** Lois Kwon, Daniela Medina, Fady Ghattas, Lilia Reyes

**Affiliations:** 1 Pennsylvania State University College of Medicine Pennsylvania State University Hershey, PA United States

**Keywords:** music, adolescent, themes, trends, primary care provider, social media, mental health, depression, anxiety, pop culture

## Abstract

**Background:**

Across the United States, the incidence of adolescent depression and suicide cases has risen in the past 10 years. Despite the risk factors and causes being multifactorial, the influence of popular culture on society and adolescents in this media-driven generation cannot be mitigated. Although the impact of social media and its effect on shaping self-identity in adolescents have been observed, the impact of music and its potential for subliminal negative messages to adolescents remains unclear.

**Objective:**

This study analyzes the lyrics and music videos of the most popular music of multiple genres to quantify the frequencies of varying music theme trends.

**Methods:**

The frequencies of themes of 1052 total American and Latin songs were collected from the Nielsen Music and Billboard’s top 100 chart performance from 1998 to 2018 for hip hop/rhythm and blues (R&B), pop, Latin, country, and rock/metal genres. Themes from songs were identified, quantified, and categorized with a rubric into negative, neutral, and positive themes by 3 different reviewers. Analysis was performed using 2-tailed *t* tests and a generalized linear model.

**Results:**

Popular songs were reviewed for positive, negative, and neutral themes in the following 3-year intervals for ease of analysis purposes: 1998 to 2000 (n=148), 2001 to 2003 (n=150), 2004 to 2006 (n=148), 2007 to 2009 (n=156), 2010 to 2012 (n= 150), 2013 to 2015 (n=150), and 2016 to 2018 (n=150). There was a significant 180% increase in the percentage of songs with negative themes between all the interval years and across all genres (*P*<.001), while there was no significant difference in the frequency of songs with positive (*P=*.54) or neutral (*P*=.26) themes by year. There were significant differences in the number of negative themes found across genres (*P*<.001), with hip hop/R&B having the highest frequency of 130 out of 208 (62.5%) of the negative themes when compared to each of the individual genres (*P*<.001).

**Conclusions:**

This study shows there is an increase in the frequency of negative themes over the span of 20 years across all genres, with hip hop/R&B having the highest frequency among the genres. These findings point to the potential impact that music may have in popular culture and on society. Furthermore, these results can help shape discussions between caregivers and their adolescent dependents and between primary care providers and their adolescent patients.

## Introduction

Overall, from 2010 to 2015, youth from the ages of 13 to 18 years in the United States were seen to have increased depressive or anxious symptoms and suicidality [1]. An observational study that investigated the relation of 38 nationally televised stories and suicide rates showed that youth seem to be at a much greater risk for imitating suicidal behavior seen on television whether fictional or true when compared to adults [2]. Although risk factors and causes are multifactorial, the influence of popular culture on society and adolescents in this media-driven generation cannot be mitigated. In fact, recent cross-sectional studies and meta-analyses have demonstrated a relationship between increased social media use and high-risk behavior among adolescents and the development of depressive symptoms [3,4]. Although the impact of social media and its effect on shaping self-identity in adolescents have been observed [5], the impact of music has not been studied.

Historically, music has influenced adolescents in the United States. For instance, new musical genres emerged during the countercultural movement of the 1960s that embodied the movements related to the conflicts in Vietnam, Civil Rights, and Women’s Rights. Musical genres emerged as a direct outlet for youth’s experimentation and need for increased liberalization through creative speech made through music. Rock and Roll music of the 1950s that originated from blues, folk, and country eventually diverged into different styles by the mid 1960s, including pop, folk, acoustic rock, and electronic music. The origins of hip hop/rhythm and blues (R&B) stem from the social injustices minorities face on a day-to-day basis that have resulted in a musical genre that uses metaphors as an outlet for African Americans and how they have come to conceptualize their environment and neighborhoods [6]. Pop music had previously embodied elements from the rock and roll of the 1950s and 1960s but eventually evolved to appeal to audiences in a way that was more “commercial, ephemeral, and accessible” [7].

Past studies have investigated how individuals employ music to induce specific emotional states in everyday situations for the purpose of emotion regulation [8]. From this perspective, the songs that receive hundreds of millions of views and the themes that their lyrics carry can potentially have unpredictable influences on adolescents. Prior research indicates that the function of musical preference as a form of identification in which adolescents can use to express their own self-concepts is indicative of the power music culture has on our youth [9]. Furthermore, recent studies are increasingly showing how music can impact interpersonal relationships, identity, agency, and emotional field in adolescent development and mental health [10].

With these factors in consideration, it is important to note that in the past two decades, hip hop and R&B music have become increasingly more prevalent and popular according to the year-end Nielsen reports, which ranked hip hop/R&B as the most-consumed genre in the years 2017 and 2018. With the rise of social media platforms such as YouTube, which amasses over 1.8 billion users a month, the medium in which music can be portrayed has diversified. Music video formats are now popular among adolescents, and studies show that music lyrics that are visually illustrated can have potentially magnified impacts [11].

The purpose of this study was to analyze the lyrics of the most popular music of multiple genres and themes, along with their respective official music videos, to capture the trends in music.

## Methods

Data were collected by reviewing the sales data across the genres of hip hop/R&B, pop, rock/metal, country, and Latin music from Nielsen Music and Billboard’s Top 100 chart performance within the United States. This study was submitted to the Institutional Review Board at Pennsylvania State Hershey Medical Center, which deemed the study to be exempt from ethical approval and informed consent, as our research did not include human participants.

The top 10 year-end songs from each genre from 1998 to 2018 were selected and analyzed in 3-year intervals for ease of statistical analysis. Frequency of themes in songs and music videos of hip hop/R&B (n=208), pop (n=212), Latin music (n=209), country (n=210), and rock/metal (n=211) were collected from 1998 to 2018 and categorized using a rubric [12] into negative, neutral, and positive themes.

A total of 46 themes were identified across all genres, with 11 positive, 21 neutral, and 14 negative themes. Themes were categorized based on the potential negative or positive influence certain topics could have on adolescent mental health. All other themes found to have ambiguous messages or influences were categorized as neutral. Negative influencers included topics such as drugs, weapons or violence, self-harm, suicide or death, crime, stigmatized mental health issues, abuse or domestic violence, discrimination or racism, alcohol use, gang participation, police brutality, misogyny, infidelity, and objectification. Neutral influencers included topics such as sexual innuendos, sexual freedom, fear or paranoia, seduction, need of help/support/guidance, loneliness, nostalgia, insecurity, insomnia, betrayal or hurt, vengeance, grief or loss, sex appeal or nudity, love, wealth, vanity, fame, partying or dancing, escapism, social injustice, and expression of emotions without enactment of anger or rage. Positive influencers included topics such as self-acceptance, empowerment or independence, homosexuality, faith, happiness, staying true to oneself, resilience, hope or strength, working hard, achieving dreams or dreaming, and growth or maturity.

Songs and music videos made available through YouTube were analyzed by 3 different reviewers by genre using a shared rubric. The rubric was developed as lyrics and videos were viewed until thematic saturation was reached. Discussions among the reviewers were regularly held concerning categorizations of certain themes as questions and concerns were raised throughout the data collection process.

In the analysis of the results, 2-tailed *t* tests and generalized linear models were used to find significant differences among years and among genres within each negative, positive, or neutral theme. Descriptive statistical analysis was also performed to look at the mean, median, and mode of positive, negative, and neutral themes for each category. The reliability between 2 reviewers was considered to be κ=0.61.

## Results

Popular songs from genres in the following time frames between 1998 and 2018 (N=1052) were reviewed for positive, negative, and neutral themes in 3 to year intervals for ease of statistical analysis. 1998 to 2000 (n=148), 2001 to 2003 (n=150), 2004 to 2006 (n=148), 2007 to 2009 (n=156), 2010 to 2012 (n= 150), 2013 to 2015 (n=150), and 2016 to 2018 (n=150). There was a significant 180% increase between all interval years in the percentage of songs with negative themes from 1998 to 2018 across all genres (*P*<.001; Table 1), but there was no statistical significance in that of the positive (*P*=.54) or neutral themes (*P*=.26; 1998 to 2000: 20.95% and 91.22%; 2001 to 2003: 28% and 84.67%; 2004 to 2006: 23.65% and 83.78%; 2007 to 2009: 24.36 and 84.62%; 2010 to 2012: 22% and 82%; 2013 to 2015: 26% and 83.33%; 2016 to 2018: 18.67% and 88.67%; Table 1). When the same parameters were used to analyze frequency of themes in music videos, there was no significant difference between the interval years in the frequency of negative (*P*=.15) or positive themes (*P*=.24; Table 2).

**Table 1:** Frequency of negative, neutral, and positive themes in lyrics based on 3-year intervals.

Period	Total themes, n	Negative themes, n (%)	Neutral themes, n (%)	Positive themes, n (%)
1998-2000	148	25 (16.89)	135 (91.22)	31 (20.95)
2001-2003	150	51 (34.00)	127 (84.67)	42 (28.00)
2004-2006	148	54 (36.49)	124 (83.78)	35 (23.65)
2007-2009	156	52 (33.33)	132 (84.62)	38 (24.36)
2010-2012	150	58 (38.67)	123 (82.00)	33 (22.00)
2013-2015	150	66 (44.00)	125 (83.33)	39 (26.00)
2016-2018	150	70 (46.67)	133 (88.67)	28 (18.67)

**Table 2:** Frequency of negative, neutral, and positive themes in music videos based on 3-year intervals.

Period	Total themes, n	Negative themes, n (%)	Neutral themes, n (%)	Positive themes, n (%)
1998-2000	148	8 (5.41)	42 (28.38)	1 (0.68)
2001-2003	150	17 (11.33)	45 (30.00)	6 (4.00)
2004-2006	148	22 (14.86)	45 (30.41)	2 (1.35)
2007-2009	156	24 (15.38)	24 (15.38)	1 (0.64)
2010-2012	150	19 (12.67)	18 (12.00)	2 (1.33)
2013-2015	150	21 (14.00)	19 (12.67)	2 (1.33)
2016-2018	150	18 (12.00)	32 (21.33)	4 (2.67)

**Figure 1:**
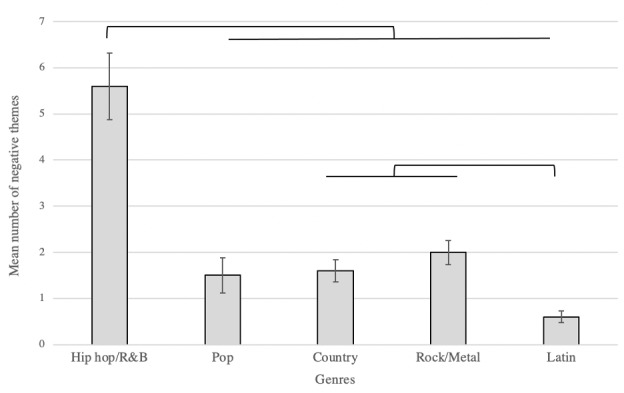
Mean number of negative themes in each genre from 1998 to 2018. Error bars reflect SE. Results show hip-hop/R&B had a significantly higher number of negative themes in lyrics when compared to other genres (*P*<.001). Additionally, Latin music significantly differed from country (*P*=.03) and rock/metal (*P*<.001). R&B: rhythm and blues.

**Figure 2:**
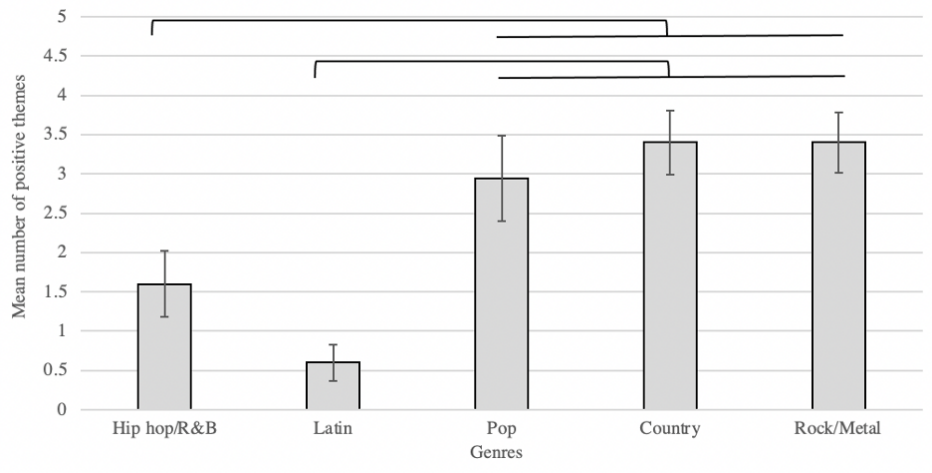
Mean number of positive themes in each genre from 1998 to 2018. Error bars reflect SE. Results show hip hop/R&B had a significantly lower number of positive themes in lyrics when compared to pop (*P*=.03), country (*P*=.003), and rock/metal (*P*=.002). Additionally, Latin music was also significantly different from pop, country, and rock/metal (all *P* values <.001). R&B: rhythm and blues.

There were significant differences found among the genres of hip hop/R&B, pop, country, rock/metal, and Latin music in the number of negative, positive, and neutral themes (all *P* values <.001; Table 3). An analysis of the songs that had at least one negative theme indicated that the number of negative themes differed between genres (*P*<.001; Figure 1). Specifically, when each genre was compared to all other genres, hip hop/R&B had a significantly higher frequency of negative themes when compared to all other genres (*P*<.001; Figure 1). Pop, country, and rock/metal had a significantly higher frequency of positive themes when compared to hip hop/R&B (*P*=.03, *P*=.003, and *P*=.002, respectively; Figure 2) and Latin music (*P<*.001; Figure 2). The thematic frequency of “drugs” and “alcohol” were highest in hip hop/R&B (Table 4).

**Table 3:** Frequency of negative, neutral, and positive themes in lyrics based on genre.

Genre	Total themes, n	Negative themes, n (%)	Neutral themes, n (%)	Positive themes, n (%)
Hip hop/R&B^a^	208	130 (62.50)	188 (90.38)	21 (10.10)
Pop	212	51 (24.06)	180 (84.91)	44 (20.75)
Country	210	76 (36.19)	172 (81.90)	73 (34.76)
Rock/metal	211	84 (39.81)	148 (70.14)	91 (43.13)
Latin	209	35 (16.75)	209 (100.00)	17 (8.13)

^a^R&B: rhythm and blues.

**Table 4:** Frequencies of themes within each genre.

Themes	Hip hop/R&B^a^, n (%) (n=208)	Pop, n (%) (n=212)	Country, n (%) (n=210)	Rock/metal, n (%) (n=210)	Latin, n (%) (n=209)
Drugs	59 (28.37)	16 (7.55)	8 (3.81)	18 (8.53)	1 (0.48)
Weapons/violence	32 (15.38)	5 (2.36)	3 (1.43)	13 (6.16)	1 (0.48)
Sex/sexual innuendos	92 (44.23)	36 (16.98)	21 (10.00)	18 (8.53)	63 (30.14)
Misogyny	12 (5.77)	0 (0.00)	1 (0.48)	2 (0.95)	0 (0.00)
Love/relationship	63 (30.29)	125 (58.96)	105 (50.00)	74 (35.07)	121 (57.89)
Wealth	88 (42.31)	18 (8.49)	1 (0.48)	6 (2.84)	2 (0.96)
Self-acceptance	1 (0.48)	5 (2.36)	3 (1.43)	2 (0.95)	1 (0.48)
Empowerment/independence	7 (3.37)	13 (6.13)	6 (2.86)	10 (4.74)	1 (0.48)
Homosexuality	0 (0.00)	2 (0.94)	0 (0.00)	0 (0.00)	0 (0.00)
Self-harm	0 (0.00)	0 (0.00)	0 (0.00)	3 (1.42)	0 (0.00)
Objectification	24 (11.54)	4 (1.89)	20 (9.52)	2 (0.95)	0 (0.00)
Alcohol	63 (30.29)	25 (11.79)	62 (29.52)	12 (5.69)	16 (7.66)
Suicide/death	7 (3.37)	0 (0.00)	0 (0.00)	36 (17.06)	0 (0.00)
Gang	15 (7.21)	0 (0.00)	1 (0.48)	1 (0.47)	0 (0.00)
Vanity	36 (17.31)	11 (5.19)	0 (0.00)	5 (2.37)	0 (0.00)
Sexual freedom	11 (5.29)	1 (0.47)	0 (0.00)	7 (3.32)	3 (1.44)
Faith	1 (0.00)	1 (0.47)	25 (11.90)	10 (4.74)	1 (0.48)
Infidelity	15 (7.21)	9 (4.25)	0 (0.00)	5 (2.37)	17 (8.13)
Fear/paranoia	5 (2.40)	2 (0.94)	0 (0.00)	6 (2.84)	0 (0.00)
Seducer	11 (5.29)	1 (0.47)	0 (0.00)	1 (0.47)	0 (0.00)
Fame	12 (5.77)	3 (1.42)	0 (0.00)	8 (3.79)	0 (0.00)
Happiness	0 (0.00)	4 (1.89)	16 (7.62)	10 (4.74)	12 (5.74)
Crime	4 (1.92)	1 (0.47)	0 (0.00)	1 (0.47)	0 (0.00)
Need of help/support/guidance	4 (1.92)	13 (6.13)	3 (1.43)	20 (9.48)	0 (0.00)
Loneliness	3 (1.44)	3 (1.42)	21 (10.00)	18 (8.53)	5 (2.39)
Partying/dancing	19 (9.13)	15 (7.08)	19 (9.05)	6 (2.84)	35 (16.75)
Nostalgia	1 (0.00)	2 (0.94)	18 (8.57)	5 (2.37)	15 (7.18)
Stigmatized mental health issues	3 (1.44)	0 (0.00)	0 (0.00)	10 (4.73)	0 (0.00)
Insecurity	4 (1.92)	4 (1.89)	0 (0.00)	6 (2.84)	0 (0.00)
Staying true to oneself	5 (2.40)	3 (1.42)	3 (1.43)	19 (9.00)	0 (0.00)
Resilience	2 (0.96)	5 (2.36)	12 (5.71)	21 (9.95)	2 (0.96)
Abuse/domestic violence	2 (0.96)	1 (0.47)	1 (0.48)	4 (1.90)	1 (0.48)
Betrayal/hurt	0 (0.00)	2 (0.94)	33 (15.71)	17 (8.06)	61 (29.19)
Hope/strength	4 (1.92)	10 (4.72)	2 (0.95)	14 (6.64)	0 (0.00)
Escapism	1 (0.48)	4 (1.89)	1 (0.48)	7 (3.32)	0 (0.00)
Vengeance	1 (0.48)	2 (0.94)	0 (0.00)	1 (0.47)	0 (0.00)
Grief/loss	2 (0.96)	5 (2.36)	21 (10.00)	11 (5.21)	24 (11.48)
Police brutality	0 (0.00)	1 (0.47)	0 (0.00)	1 (0.47)	0 (0.00)
Discrimination/racism	3 (1.44)	1 (0.47)	0 (0.00)	1 (0.47)	0 (0.00)
Working hard	4 (1.92)	1 (0.47)	8 (3.81)	6 (2.84)	0 (0.00)
Achieving dreams/dreaming	7 (3.37)	5 (2.36)	13 (6.19)	7 (3.32)	0 (0.00)
Sex appeal/nudity	0 (0.00)	0 (0.00)	0 (0.00)	1 (0.47)	8 (3.83)
Growth/maturing	1 (0.48)	0 (0.00)	7 (3.33)	18 (8.53)	0 (0.00)
Social injustice	0 (0.00)	0 (0.00)	0 (0.00)	5 (2.37)	0 (0.00)
Expressive emotions: anger/rage	0 (0.00)	0 (0.00)	0 (0.00)	3 (1.42)	0 (0.00)

^a^R&B: rhythm and blues.

**Figure 3:**
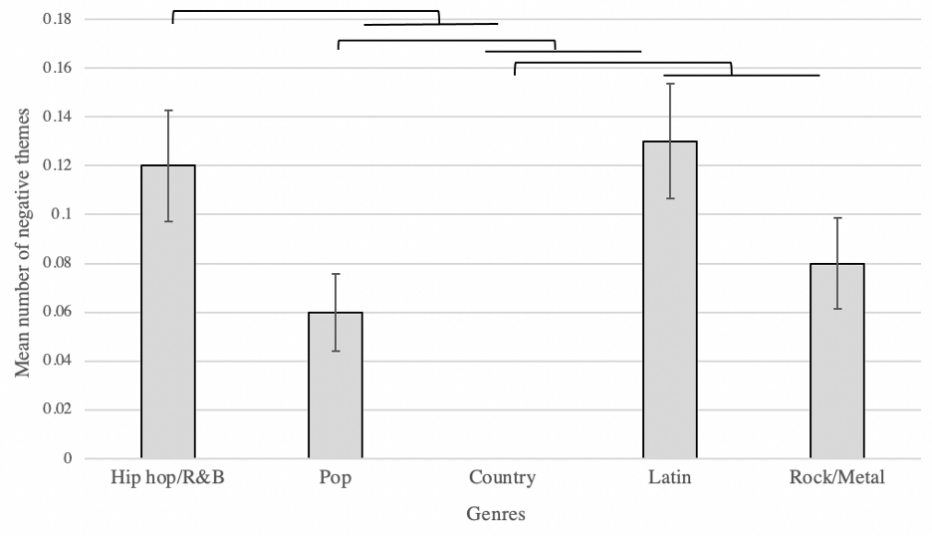
Mean number of negative themes in each genre from 1998 to 2018. Error bars reflect SE. Results show hip hop/R&B had a significantly higher number of negative themes in music videos when compared to pop (*P*=.02) or country (*P*<.001). Pop had a significantly higher number of negative themes when compared to country (*P*<.001) or Latin music (*P*=.01). Country had a significantly higher number of negative themes when compared to Latin music (*P*<.001) and rock/metal (*P*<.001). R&B: rhythm and blues.

There were significant differences in the number of negative (*P*<.001) and neutral (*P*<.001) themes found among genres in music videos (Table 5), with hip hop having the highest number of negative themes portrayed (*P*=.001; Figure 3). There was no significant difference in the number of positive themes found among genres in music videos (*P*=.10; Table 5).

**Table 5:** Frequency of negative, neutral, and positive themes in music videos based on genres.

Genre	Total themes, n	Negative themes, n (%)	Neutral themes, n (%)	Positive themes, n (%)
Hip hop/R&B^a^	208	58 (27.88)	54 (25.96)	3 (1.44)
Pop	212	19 (8.96)	41 (19.34)	5 (2.36)
Country	210	0 (0.00)	0 (0.00)	0 (0.00)
Rock/Metal	211	19 (9.00)	48 (22.75)	7 (3.32)
Latin	209	32 (15.31)	81 (38.76)	3 (1.44)

^a^R&B: rhythm and blues.

## Discussion

This study shows that there has been a significant increase in the frequency of negative themes over the span of 20 years across all genres, with hip hop/R&B having the most the most number of negative themes among all other genres. Pop, country, and rock/metal had significantly higher frequencies of positive themes when compared individually to hip hop/R&B and Latin music. The frequency in which these negative themes are repeated in a particular song is not trivial but may contribute to normalizing certain activities or attitudes of themes, such as the use of drugs, weapons, or alcohol.

There was also a significant difference in the number of negative themes found among genres in music videos, indicating that certain genres visually portray these negative themes more so than others. With the recent rise in popularity of music video streaming platforms, such as YouTube, themes relayed through lyrics can potentially magnify the messages delivered in a song.

Music has historically played a multifaceted role by serving as a form of creative expression to convey emotions or as a constructive outlet for individuals to explore feelings through direct engagement. It important to note that the potential social psychological impacts of music cannot be mitigated, as music has become a pervasive part of society, especially for youths who live in a world saturated with media. In fact, with the diversity of musical genres, individuals have developed stereotypes of those who listen to particular musical genres [13]. Moreover, music has been found to influence “intergroup dynamics by shaping in-groups and out-groups” based on musical preferences [14].

Given that the average youth encounters up to 8 hours’ worth of media content [15], adolescents are potentially exposed to pop culture more, which therefore makes music a common experience of the average US teenager and is worth exploring further.

Adolescents are at a particularly impressionable age when environmental and social encounters can largely shape identity formation. Therefore, the results from this study should be noted when considering the probable ramifications of increased frequency of negative themes in both lyrical content and visual representation of music, especially those of the hip hop/R&B genre. Findings from this study can be used to guide recommendations given by parents, educators, and providers to discuss with adolescents.

There are important limitations to take into consideration. As only the top songs of each year from each genre were analyzed over the span of 20 years, making broad, generalized conclusions about a specific genre is not possible. Furthermore, there is also no causal inference that can be made concerning the findings, as this study only analyzed content and thus lacks evidence to quantify the impact on mental health. Future studies should potentially involve surveying adolescents to more accurately identify the popular music listened to by this age group, which may not be necessarily reflected in the year-end top songs.

Another limitation is the accuracy in categorization of some of the themes found within certain songs by each reviewer. Although discussions were extensively held regarding the labeling of themes and the positive, neutral, and negative categories in which we grouped them, songs frequently conveyed messages without using explicit terms through the use of analogies or wordplay that could have made the interpretation more ambiguous. Some themes such as anger or rage were categorized as neutral, which may seem controversial, but efforts were made to fully elucidate the way in which these emotions were presented in a song. If a particular song focused solely on the emotion itself, we viewed this as a neutral theme, as it is an expressive emotion that humans experience. However, when these feelings resulted in a violent act or depressive thought, themes were classified as such within the negative category. Additionally, reviewers were adults who might have interpreted certain musical lyrics and images differently from adolescents. Future studies should consider including the perspective of adolescents when categorizing musical themes. Surveys could also help to identify additional media sites that are popular among adolescents.

This study explores the trends in music in the past 20 years by quantifying the frequency at which certain themes arise. Results show that there was a significant 180% increase in the negative themes of songs of all genres (*P*<.001) but no significant difference in frequency of songs with positive (*P*=.54) or neutral (*P*=.26) themes by year. There were significant differences in the number of negative themes found across genres (*P*<.001), and hip hop/R&B had the highest frequency when compared to other genres (*P*<.001), with 130 out of 208 (62.5%) of its themes being negative. Although preliminary, these findings highlight the potential influence of music and may help to shape recommendations set by pediatricians and frame conversations held by parents with children and adolescents.

Future studies should look into increasing the number of songs analyzed per year using similar methods to increase the power of the study. Another region to explore is the influence of famous music artists on adolescents through surveys that explore the extent to which individuals have adapted their own attitudes to mirror those of their idols. Understanding how the personas of popular music artists directly affect adolescents may add an interesting dimension to future research and be used to identify those factors that have a greater correlation to mental health.

## Abbreviations

R&B: rhythm and blues
